# Welcome to Volume 8 of the *International Journal of Hematologic Oncology*

**DOI:** 10.2217/ijh-2018-0012

**Published:** 2019-02-26

**Authors:** Jennifer Straiton

**Affiliations:** 1 Future Science Group, Unitec House, 2 Albert Place, London, N3 1QB, UK

As the new Editor for the *International Journal of Hematologic Oncology*, it is with great excitement that I introduce the first issue of Volume 8. The year 2019 has lots of interesting things to be excited about; new projects, new ideas and, of course, more excellent content from all of our authors. The return of our Journal Watch series will help to broaden the range of topics we are able to publish, increasing our coverage of the latest information in the field.

However, before we start this new period I would like to look back over the past year and our highlights from 2018.

The year 2018 was a year of change for the editorial team at *International Journal of Hematologic Oncology* with new faces starting and the journal gaining recognition. Our presence on PubMed Central has been a huge help in widening our audience and increasing the reach of the articles we publish.

## Content highlights of 2018

The standard of content that we publish has remained high. This is due to both our authors and our reviewers who utilize our peer review and revisions policy to ensure all work is at its best. We have received a variety of article types from an international range of authors, each piece providing novel, interesting and exciting developments in the field of hematologic oncology management.

The most-read article of 2018 also happens to be our most-read article ever published. Yazeed Sawalha and Anjali Advani from the Taussig Cancer Institute (OH, USA) reviewed the management of acute lymphoblastic leukemia in older generations, discussing the current approaches and the challenges faced by those in this field [1]. Pointing out the poor prognosis for this age group, they stressed the need for more research in the area.

Another 2018 article that has hit our most-read list is an editorial from Pratap Neelakantan and Adam Mead, both of the University of Oxford (UK), who described the history of chronic myeloid leukemia and how treatments have developed through the last 5000 years [2]. Remaining hopeful, they ended on a positive note, suggesting that if advancements continue at this rate then a cure could be in sight.

We have also published several original research articles; one of our highlights is from the Harbin Institute of Hematology & Oncology (China), which provides an observational study of Chinese adults with relapsed/refractory Philadelphia-negative acute lymphoblastic leukemia [3]. This form of leukemia often has a poor outcome in this population group so this study from Ma *et al*. could help in providing reference material when determining the course of treatment.

### Editorial Board

To our Editorial Board, we thank you for your continued input, be it in an ambassadorial, advisory or authorship role. Led by our Senior Editor Michele Baccarani (University of Bologna, Italy), our international board provides valuable assistance and advice that facilitate the publication process; we look forward to working together in the coming year.

On this note, if you are interested in joining our advisory board or wish to provide feedback or suggestions for the journal, please do not hesitate to get in touch; we value any and all input that can contribute to the growth and development of the journal.

### Article outreach

To help further spread the work we publish, we continue to utilize the power of social media and share new work through platforms such as Twitter to ensure articles can be shared with the largest possible audience; if you don't already, we welcome you to follow us on Twitter (@fsgijh). In using social media it is our aim to reach all relevant stakeholders in the field of hematologic oncology – including researchers, clinicians, charities, patients, academics/educators and patient advocates, just to mention a few.

Our partnership with the site Oncology Central [4] continues, giving authors the opportunity to put their work on the Oncology Central website and be seen by its wide readership base. Registration to Oncology Central is free and allows you to keep up to date with the latest developments in cancer via unparalleled free access to the latest news, opinion, peer-reviewed journal articles, multimedia and exclusive content.

## Conclusion

Our readers remain central to the success of our journal and we welcome any feedback. Please do not hesitate to contact us with any suggestions for what you would like to see featured or any article proposals of your own. We welcome a wide range of unsolicited article proposals so please do get in touch for more information.

I would like to finish by once again thanking all of the authors and reviewers who helped to make Volume 7 possible; we hope to continue to build on this success and look forward to another great year.
